# Supplementary material for: “People here live in denial”: A qualitative study of the pervasive impact of stigma on asthma diagnosis and care in Kenya and Sudan
Source: PLOS Glob Public Health. 2025 Dec 16;5(12):e0003935. doi: 10.1371/journal.pgph.0003935 (PMC12707672; doi:10.1371/journal.pgph.0003935)
Supplement: S1 Text — (DOCX) [file pgph.0003935.s001.docx]

**International Multidisplinary Programme to Address Lung Health & TB in Africa(IMPALA)**

**An integrated health systems approach for improving health care services for chronic lung diseases in Sudan and Tanzania**

**Research Toolkit: Study Phase One and Two**

**FGD Topic Guide for Community Members**

FGD ID NO: ______________ Facilitator Initials: ___________ Note-taker Initials: __________

Participant group________________ Number of participants___________ Audio file ID _________

Country/Community: _____________________ Date of FGD _____________________

***Community understanding of CLD***

- What are the main diseases in this area? (Probe for reasons)

***In our discussion today, we are going to focus on lung health and chronic lung diseases such as asthma, COPD, occupational lung disease and TB.***

- What does it mean to have healthy lungs?
- What does it mean if a person does not have healthy lungs?
- In your community is there a word/ term for diseases that affect the lungs over a long time?
- If yes, what is the word/term?
- Probe what this word/term mean
- What are the general symptoms of lung health problems?
- What activities or practices in the community cause lung health problems?
- How might lung problems affect someone’s life? Probe what activities someone might experience challenges with or might not be able to do
  prope (self-care; community participation; livelihood activities; mobility)
- What are the community attitudes and perceptions towards people with chronic lung disease problems?

***Community care-seeking for CLD***

- If someone in your community has lung problems, what might he/she do to manage them?
- Probe home remedies, care seeking with traditional healer (Spiritual healer, traditional medicine, others), care seeking at health facility, go directly to the pharmacy
- Why might someone with lung problems decide to use home remedy/go to a traditional healer/ seek care at a health facility?
- Please tell me more about the services provided at the health facility
- Probe what do you like about them?
- What could be improved about the services?
- Probe trust in existing services. (availability, quality, interaction with health provider, confidentiality, treatment outcome)
- What services do you think should be available for someone with lung problems? Probe for differences between health centre and hospital, public and private
- Probe why?

***Community priorities for care for CLD***

- What would make it easier for someone with lung problems to cope? Why?
- What can be done differently to help someone manage their lung problems?
- Is there anyone in your community who has lung problems who does not attend the health facility?
- Why do you think he/she does not attend?
- What do you think would make it easier/ more helpful for that person to attend? (probe for diagnostic facilities, medicine, enough staff, good client provider relationship, cost)
- If the CHW or a health worker (trained volunteer)was able to refer someone with lung problems to the health centre/district hospital, would that make a difference? Why? Why not?
- What would you think if the health centre/district hospital were able to do a test to find out what was causing lung problems? Why?
- What would you think if the health centre/district hospital were able to write a prescription for medicine that would help someone with lung problems feel better if he/she kept taking it regularly? Why?

**Interview Topic Guide for in-depth interviews with community members (including those affected by CLD)**

IDI ID NO: ______________ Facilitator Initials: ___________ Note-taker Initials: __________

Participant group________________ Number of participants___________ Audio file ID _________

Country/Community: _____________________

Where do you work:______________________

Are you married:_________________________

Do you have children:_____________________

Date of IDI _____________________

***Community understanding of CLD***

- What does it mean to have healthy lungs?
- What does it mean if a person does not have healthy lungs?
- In your community is there a word/ term for diseases that affect the lungs over a long time?
- If yes, what is the word?
- Probe what this word means
- I understand that you have experienced lung problems. Can you tell me more about the symptoms you have? How long have you had these symptoms?
- What do you think are the main causes of your lung health problems?

***Impact of CLD on quality of life***

- How has your lung problem influenced your daily activities?
- Probe: what activities can you do/ not do; self-care; community participation; livelihood activities; mobility
- How do you feel about your lung problem?
- Probe: impact on mental wellbeing
- Who are you able to talk to about how you feel?
- Has your lung problem affected your household finances? How?
- Probe: for impact on household spending? Influence on livelihood activities (if not discussed above)? Cost of care seeking? Medicines?
- Can you tell me how you cope with the changes that your lung problem has brought to your life?
- Do you know anyone else in your community living with lung health problems?
- If yes, how do you interact with them? Are you able to talk to them about how you feel?

***Community care-seeking for CLD***

- What did you do when you started to develop these symptoms related to your lung health problem? Probe home remedies, care seeking with traditional healer, care seeking at health facility
- Why did you decide to use home remedy/go to a traditional healer/ seek care at drug shops, pharmacies or a health facility?
- (If attended health facility). Please tell me more about what happened when you went to the health facility. Probe investigations, management, perception of quality/ effectiveness
- (If attended health facility). What was good or bad about the services you got at the health facility? Probe trust in services
- (If didn’t attend health facility). Why did you decide not to attend health facility?
- (If didn’t attend health facility). What would make you more likely to attend the health facility for your lung problems? Why?
- Why would people with lung disease not attend the health facility?
- What do you think would make it easier/ more helpful for them to attend?

***Community priorities for care for CLD***

- What services do you think should be available for someone with lung problems? Probe why?
- Is there anything else that could be done to help you?
- Probe for: physically, mentally, socially, medically
- Why?
- If someone is trained in your community to know the signs and symptoms of CLD and has the capacity to refer you to the nearest health facility, would that help? Why? Why not?
- What would you think if the health centre/district hospital was able to do a test to find out what was causing your lung problems? Why?
- What would you think if the health centre/district hospital was able to write a prescription and provide medicine that would help you feel better if you kept taking it regularly? Why?

**Community experiences with community TB services (Tanzania only)**

- (Tanzania only). Have you told your CHW about your lung problem? Why? Why not?
- (Tanzania only). Can the CHW help you to get health services for your lung problem? Why? Why not?
- (Tanzania only). How could the CHW help you more with your lung problem?

26. What would you think about CLD services being integrated with TB services? Would you be willing to receive care through the TB programme? Why/Why not?

Interview Topic Guide for Health Workers at EPILAB Sites

Interview ID NO: ______________ Facilitator Initials: ___________ Note-taker Initials: ________

Type of Participant ________________ Audio file ID _________

Health facility: _____________________ Date of Interview ___________________

Where do you work? ____________________________

What is your job title? _________________________________________________

How long have you worked as (job title)? __________

***Level of priority for CLD services***

- Can you tell me more about the services that you provide at this health facility?
- What are the main priority diseases and illnesses for this facility?
- Why are these the main priorities?

***Strengths and weaknesses of CLD integration***

- Can you tell me more about the services you provide for patients with asthma at this facility?
- What algorithms or processes do you follow? Can you show me?
- What does effective asthma care look like?
- How is this documented? Can you show me the documents?
- Can you tell me about the integration of asthma management services within your health facility?
- What is good about integration of asthma management services?
- What is bad about integration of asthma management services?
- What challenges have experienced in providing care for patients with asthma?
- How could these services be improved?
- Can you tell me more about how you would diagnose a patient with asthma?
- What would make it easier for you to diagnose a patient with asthma?
- Prompt for: equipment used, use of algorithms, use of referral systems,
- Can you tell me more about how you would manage a patient with asthma?
- What would make it easier for you to manage a patient with asthma?
- Prompt for: medicines used and their availability, use of registers, out-patient clinics (frequencies/staff involved etc).

***Pathways towards improvement for CLD services***

- Can you tell me more about the availability of health workers at this facility?
- Which of these health workers support diagnosis and management of patients with asthma? How do they support these patients?
- Can you tell me more about the supervision structure for these health workers?
- How could supervision be improved?
- Are there any other health workers that you think should be involved in caring for patients with asthma, who are not currently part of the asthma management team? Please tell me more
- Can you tell me more about the availability of equipment for diagnosing asthma at this facility?
- How could this be improved?
- Can you tell me more about the availability of drugs to manage asthma at this facility?
- How could this be improved?
- How accessible are these drugs to patients?
- Prompt for: stock outs, cost etc.
- Can you tell me more about how you collect data for patients with asthma at this facility?
- How could this be improved?
- How is this used in decision making?
- Who do you share this data with? How often is it shared?
- What do you think are the main needs for patients with asthma?
- What changes need to happen at this facility to meet these needs?
- How do you think lower levels of the health system (e.g. primary health centres/community health workers) could be involved in the provision of asthma management services?

**Interview Topic Guide for known CLD Patients (EPILAB Sites)**

IDI ID NO: ______________ Facilitator Initials: ___________ Note-taker Initials: __________

Participant group________________ Number of participants___________ Audio file ID _________

Country/Community: _____________________

Where do you work:______________________

Are you married:_________________________

Do you have children:_____________________

Date of IDI _____________________

***Community understanding of CLD***

- What does it mean to have healthy lungs?
- What does it mean if a person doesn’t have health lungs?
- What did the doctor / health worker tell you about your illness / condition when you were diagnosed?

***Impact of CLD on quality of life***

- I understand that you have experienced asthma. Can you tell me more about the symptoms you have? How long have you had this condition?
- How has asthma influenced your daily activities?
- Probe: what activities can you do/ not do; self-care; community participation; livelihood activities; mobility
- How do you feel about living with asthma?
- Probe impact on mental wellbeing
- Who are you able to talk to about how you feel?
- Has your asthma affected your household finances? How?
- Probe: for impact on household spending? Influence on livelihood activities (if not discussed above)? Cost of care seeking? Medicines?
- How much money do you spend per month on accessing medicines/treatment for asthma?
- Can you tell me how you cope with the changes that asthma has brought to your life?
- Do you know anyone else in your community with the same problem as you?
- If yes, how do you interact with them? E.g. talking/sharing of medicines etc.
- Are you able to talk to them about how you feel?

***Community care-seeking for CLD***

- What did you do when you started to develop these symptoms?
- Probe home remedies, care seeking with traditional healer, care seeking at health facility
- Why did you decide to seek care at a health facility?
- Please tell me more about what happened when you went to the health facility.
- Probe investigations, management, perception of quality/ effectiveness
- What was good about the services you got at the health facility?
- What was not good about the services you got at the health facility?
- What steps do you take now when you start to experience symptoms of asthma/asthma attack?
- How often does this occur?

***Community priorities for care for CLD***

- How can the asthma management services at the health facility be made better?
- What do you think are the main priorities for health services for people with asthma?
- What would make it easier to cope with your asthma? Why?
- Probe for: physically, mentally, socially, medically
- Is there anyone in your community who has asthma who does not attend the health facility?
- Why do you think he/she does not attend?
- What do you think would make it easier/ more helpful for that person to attend?
- What could be done within your community to make asthma management easier?
- Prompt for: community support groups; links to community health workers/other health providers etc.

Informed Consent Form (All Participants)

Version 1: May 2018

This form should be administered alongside the appropriate information sheet for the study participant. If the information sheet is updated, version control to the below consent form should be applied.

| **CONSENT FORM**  Study title: IMPALA (International Multidisciplinary Programme to Address Lung Health and TB in Africa): Introducing an integrated package of care for chronic lung disease: a community health systems analysis in Sudan and Tanzania  Lead National Qualitative Researcher ______________________________________________  By signing this form, I agree that [ *tick the box as appropriate*]:   - I have received the participant information sheet (Version 1: May 2018). The study has been explained to me in a language that I understand. All the questions I had about the study have been answered. I understand what will happen and what is expected of me.   Yes No   - I have been informed that it is my right to refuse to take part in the research and that if I choose not to take part I do not have to give a reason. Yes No - I have been informed that anything I say during the research will remain completely confidential: my name will not be used nor any other information that could be used to identify me. Yes No - I agree to maintain confidentiality of information shared during this research*.*   Yes No   - I have been informed that my conversation in this research will be recorded on a digital recorder. I have been informed that I can choose to have any part of my conversation not recorded. Yes No - I am aware that due to the nature of the study topics certain sensitive information about me could be revealed to the interviewer and research team. Yes No - It has been explained to me that the researchers may use my own words when writing up the findings of the research. I understand that any use of my words would be completely anonymous (without my name). I have been told that I can decide whether I permit my words to be used in this way. Yes No   Circle your response:   \| I agree to take part in the study: \| Yes \| No \| \| --- \| --- \| --- \| \| I agree that my own words may be used anonymously in the report \| Yes \| No \| \| I agree that this conversation should be recorded on a digital audio recorder \| Yes \| No \|   **Signature of participant:**   \| **NAME**  (in capital letters) \| **SIGNATURE OR THUMB PRINT** \| **DATE OF SIGNATURE**  (in DD/MM/YYYY) \| \| --- \| --- \| --- \| \|  \|  \|  \|   **If a thumb print is provided, signature of witness:**   \| **NAME**  (in capital letters) \| **SIGNATURE** \| **DATE OF SIGNATURE**  (in DD/MM/YYYY) \| \| --- \| --- \| --- \| \|  \|  \|  \|   Tick box if participant refuses to have witness present  **Signature of study staff taking consent:**  I have discussed the study with the respondent named above, in a language he/she can comprehend.  I believe he/she has understood my explanation and agrees to take part in the interview.   \| **NAME**  (in capital letters) \| **SIGNATURE** \| **DATE OF SIGNATURE**  (in DD/MM/YYYY) \| \| --- \| --- \| --- \| \|  \|  \|  \| \|  \|  \|  \|   ***NOTE:*** *A copy of the signed, dated consent form must be given to the participant.* |
| --- | --- | --- | --- | --- | --- | --- | --- | --- | --- | --- | --- | --- | --- | --- | --- | --- | --- | --- | --- | --- | --- | --- | --- | --- | --- | --- | --- | --- | --- | --- |
