# Supplementary material for: “People here live in denial”: A qualitative study of the pervasive impact of stigma on asthma diagnosis and care in Kenya and Sudan
Source: PLOS Glob Public Health. 2025 Dec 16;5(12):e0003935. doi: 10.1371/journal.pgph.0003935 (PMC12707672; doi:10.1371/journal.pgph.0003935)
Supplement: S2 Text — (DOCX) [file pgph.0003935.s002.docx]

Chronic respiratory diseases in Meru County, Kenya: how do public health systems respond and what are the opportunities for health system strengthening?

**Appendix 1_ FGD COMMUNITY MEMBERS**

**FGD ID NO: ______________**

**Date**

**Duration**

1. Please tell me about the common health problems in this community?
2. When people fall ill, where do they go to first (probe: shop, traditional herbalist, health centre, private health facility, hospital).
3. For each of these places, why do you think people decide to go there first?

**Chronic respiratory diseases**

1. Now I want us to discuss about health problems affecting the chest ***(describe- these problems are for example, difficulty in breathing, pain in chest, coughing a lot, breathing that produces some sound. These problems take more than two weeks, some people even live with these conditions for long).*** How common are these problems in this area?
2. Have you ever experienced any these problems yourself? Please describe how it was?
3. Have you ever seen anyone in the community with any of these problems? (probe: family member, child, neighbour, relative)
4. What actions do people take, when they experience these problems with the chest? (probe: where do they go for treatment? Probe: shop, traditional herbalist, health centre, private health facility, hospital)
5. Please tell me your experiences when using healthcare facilities that are run by the county government (what do you like most about the services for the chest problems?- probe: availability of medicine, tests- lab, chest x-ray, healthcare workers, duration of services).
6. Have you ever been asked to take any test for chest problems? (probe: sputum, Chest x-ray) Please describe how you did it? Did you pay anything? How long did it take to get the results? After results what did the doctor say?
7. What do you **not** like most, when you go to these facilities (availability of medicine, tests- lab, chest x-ray, healthcare workers, duration of services).
8. Has anyone ever come to your homes to talk to you about health? What do they talk about? Have they ever talked about these chest problems? Have they ever asked you to go to healthcare facilities?
9. Are there any groups/ organizations within this community that talk to community members about their health? How do they do it?
10. How can services in our healthcare facilities be improved, to take care of people with problems such as coughing, chest problems?

**Appendix 2_ IDI/ FGD GUIDE COMMUNITY HEALTH VOLUNTEERS**

**IDI NO: ______________**

**Date**

**Duration**

1. Please describe your roles in supporting healthcare in this community.
2. How many CHV are in your team?
3. I wish to understand more about chronic lung diseases (*Term used by the CHEW will be used here*). What do you think CRD means? Who gets CRD? What symptoms would they have?
4. Please explain what your work involves in helping community members with cough and breathlessness who do not have TB (Probe: awareness creation, how would you identify someone? Do you differentiate cases with TB from those with a cough but do not have TB? How do you refer of CRD cases? Who do you refer them to?).
5. How regularly do you communicate with the CHEWs about a patient with CRD that you have referred for care? How do you know if patient received care at the facility? Are there times that patients who you refer to facilities fail to receive healthcare (probe: diagnosis, treatment, any service at all)? In such instances, what actions do you take?
6. After a patient comes from the health facility, what happens next? (probe: do you follow up patients with chest problems, at their homes? Do you ever involve family members in caring for patients? Do you have patient supporters for any other disease, apart from TB?)
7. Have you ever received a call from a sub county hospital about a patient who comes from this community? What was the call about?
8. Did you receive any training on CRD management? When did you receive the training? Who provided this training?
9. What has worked well so far? Why do you say so?
10. What has not worked well? Why do you say so?
11. In your view, what should be done to improve your work as a CHV?

**Appendix 3_ Indepth Interview COMMUNITY HEALTH EXTENSION WORKER**

**IDI NO: ______________**

**Date**

**Duration**

1. Please tell me about your role as a CHEW. How many households do you support in this area?
2. Is chronic respiratory disease an issue in this CHU? What are the main chronic respiratory diseases affecting people in this community? What is your involvement in CRD in your CHU?
3. Please describe how referrals for people with CRD to this facility works (probe: how patients are referred, any documentation from the CHV, records kept by CHEW; coordination between the community and hospital care systems).
4. How frequently are CRD patients referred? How does it work? What is your involvement?
5. After initial treatment, how do you follow up with patients’ treatment? *(Probe: how do you get feedback about patients’ condition, supporting follow up, self-management)*
6. Do you have any tobacco cessation programs in in your community? How is it working?
7. Did you receive any specific training on CRD management? (If yes, when? Do you have any guidelines for supporting your community work?
8. Have the CHV been trained in CRD case detection? Please tell me about module 13 of the community health strategy? Have you been trained on it? Have CHV been trained? How is it working?)
9. Apart from hospital care, what other support do you provide patients in the community? Do you conduct any community outreach services on CRD? How frequently do you do this? What is involved in the outreach? How do you involve household members in the care of the patients? How is this working?
10. What form of support do you receive from the county government in the community health system? (Probe: financing, advocacy, technical support) Apart from county government, who are the other partners that you work with? (Probe: international organizations, local organizations)
11. What has worked well so far? Why do you say so?
12. What has not worked well so far? Why do you say so? What could be done differently to improve partnership between the health facility and community?

**Appendix 4 In-depth interview for healthcare workers: clinical staff e.g. nurse, CLD specialist**

**IDI NO: ______________**

**Date**

**Duration**

1. Please describe to me your roles in this department (probe: how long have you been in this role? How big is your team? Please describe their roles?)
2. Do you see patients with chronic respiratory diseases? What conditions do you mostly see? How would you know if someone has CRD? What challenges do you face in diagnosing CRD?
3. What makes people with chronic respiratory symptoms come here? (probe- are they referred here? From where? Who refers them?) Please describe what happens to these patients from the time they come in. (Probe: Screening and diagnostics, treatment, follow up, health education).
4. Do you have access to specialists to support your decision-making in management of chronic respiratory diseases? Please describe how it is working here?
5. How does referral work here? Where are patients referred from? Where to? How frequently do you refer patients? After referral, do you maintain contact with the patient? How? Do you get feedback? What happens after discharge?
6. Please tell me what has worked well so far in diagnosis of CRD patients
7. Please tell me what has **not** worked well in diagnosis of CRD patients. (For each of the observations, why do you say so?)
8. Please tell me what has worked well so far in treatment of CRD patients.
9. Please tell me what has **not** worked well in treatment of CRD patients. (For each of the observations, why do you say so?) Probe availability of drugs, timeliness of county governments, affordability of drugs, follow ups).
10. How do you ensure continuity of care for people with chronic respiratory diseases, after they leave this facility? (probe: in what ways do you support self-management of patients?)
11. Did you receive any training specific to CRD patients? When was that? (Probe: use of spirometers to diagnose chronic lung diseases) Do you have guidelines specific to CRD management? What are your experiences in implementing those guidelines?
12. What are your views of how services for CRD can be improved?

**Appendix 5_ Indepth Interview guide for pharmacy staff**

**IDI NO: ______________**

**Date**

**Duration**

1. Please describe to me your roles in this pharmacy department (how long have you been in this role? How big is your team? Please describe their roles?)
2. I am interested in understanding the process of treatment for patients who come with chronic respiratory problems. Please describe how these patients get services from the pharmacy. (Probe: prescriptions, drugs access, demonstrations of using drugs/ inhalers?).
3. Please tell me about the preferred drugs for CRD. Which of these do you stock? How do you procure these drugs? What are your experiences of availability of these drugs? (probe: How frequently do you run out of stock? How long? what happens when stocks out?)
4. How do you manage your stocks? Do you use any electronic system? Please explain how this works? How does it work?
5. What is your experience about use of NHIF by patients to pay for their medication? What happens if a patient cannot pay for their medicine?
6. Apart from drugs, do you procure any equipment used in the management of CRD e.g. spacers?
7. Please tell me what has worked well so far in treating CRD patients.
8. Please tell me what has not worked well in treating CRD patients. *(Probe For each of the observations, why do you say so? availability of drugs, timeliness of county governments, affordability of drugs, follow ups).*
9. Did you receive any training on CRD management? (If yes: when?)
10. Do you have guidelines specific to CRD management? What are your experiences in implementing those guidelines?
11. What are your views of how services for CRD can be improved in this county?

**Appendix _7_ Indepth Interview guide for health facility Managers**

**IDI NO: ______________**

**Date**

**Duration**

**Appendix 3_m; In-depth interview for Facility in charges and senior management staff**

1. Please describe to me your roles in this department (probe: how long have you been in this role? How big is your team? Please describe their roles?)
2. What conditions of chronic lung disease do you mostly see? How would you know if someone has CLD? What challenges do you face in diagnosing CLD?
3. What makes people with chronic respiratory symptoms come here? (probe- are they referred here? From where? Who refers them?) Please describe what happens to these patients from the time they come in. (Probe: Screening and diagnostics, treatment, follow up, health education).
4. Does this facility have access to specialists to support your decision-making in management of chronic respiratory diseases? Please describe how it is working here?
5. How does referral work here? Where are patients mostly referred from? Where to? How frequently do you refer patients? After referral, do you maintain contact with the patient? How? How do you get feedback on patient condition? What happens after discharge?
6. Please tell me what has worked well so far in diagnosis of CRD patients? Probe- Please tell me what has **not** worked well in diagnosis of CRD patients. (For each of the observations, why do you say so?)
7. Please tell me what has worked well so far in treatment of CRD patients? (For each of the observations, why do you say so?) Probe availability of drugs, timeliness of county governments, affordability of drugs, follow ups). Please tell me what has **not** worked well in treatment of CRD patients.
8. How do you ensure continuity of care for people with chronic respiratory diseases, after they leave this facility? (probe: in what ways do you support self-management of patients? Do you have treatment supporters for patients with chronic lung conditions?)
9. Please describe how you prioritize which supplies should be brought to the facility. (Probe: how do you negotiate for essential supply for supporting the facility? Do you always get what you ask for? Please explain instances where your requisitions are not authorized)
10. In what ways has NHIF supported care of people with chronic lung conditions? How do you process claims for people who have chronic conditions? (probe- do the payments come to the facility directly, or they are channeled to the county)Are there services that are excluded? What are the caps to NHIF reimbursements for CLD?
11. What is your experience in enhancing staff capacity to diagnose chronic lung diseases? (probe: do you use guidelines? do you offer in-service training to healthcare workers)
12. What is your experience in enhancing staff capacity to manage chronic lung diseases? (probe: do you use guidelines to support management of diseases? do you offer in-service training to healthcare workers)
13. In what ways do you foster linkage between your facility [and the community health system (for health centre in-charges) ] ; partnerships with other community partners/ stakeholders?
14. In what ways do you foster linkage between your facility and lower level facilities; higher level facilities (for hospital managers); partners with other community partners/ stakeholders?
15. Do you have smoking cessation programs in this facility? Please describes how it is working? Please describe challenges you face in running the smoking cessation program
16. What has worked really well in this county in so far as chronic lung diseases management is concerned?
17. What has not worked well in this county in so far as chronic lung diseases management is concerned?

**Appendix _8_ Indepth Interview guide for patients**

**IDI NO: ______________**

**Date**

**Duration**

1. Please tell me why you came to this (health centre/ hospital)
2. When did you (or child) start feeling bad?
3. What did you do to relieve the bad feeling/ pain? (probe: took medication- what was the source of medication? E.g. shop, from friend/relative/ traditional herbs). Apart from taking medicine, what other action did you take? (probe: prayers, health facility).
4. Is this the first time you are coming to this facility for this problem?
5. If yes in 4 above- (did you visit any other facility before you came here?). when? What did they tell you the problem was? Were you given medication? How did you feel?
6. If not, when did you come last?

**Experiences in this facility**

I want to now discuss about your experiences in getting healthcare services in this facility. Please tell me all you know, because as I said, the information that you will provide is important in helping to improve services here, and other parts of Kenya.

1. For today’s visit, what did the doctor tell you is the problem? Did they write somewhere? Which tests did you take? Have you received the results? If not, when have you been asked to come back for results?
2. For this problem, what is the next step for you? (probe: have been given medicine, follow up date? When? Have been asked to go for other tests; have been referred to another hospital?)
3. *For those given medicine:* what medicine have you been given? Did you get the medicine from the hospital pharmacy or have you been asked to buy? Have you bought the drug? if not why? (If bought, have you been told what to do when you get at home? Please explain a bit?)
4. How are you paying for the services here? (probe: consultation, lab, x-ray, drugs). Do you have NHIF? Have you used it in buying drugs here? Have you ever used NHIF in this hospital?
5. Have you been told to talk to anyone else after you leave here?
6. Do you smoke? If yes, have you been told anything about smoking? (probe: have you been told anything about stopping to smoke? What have you been told?)
7. What did you like most about the services you received in this facility today?
8. What did you ***not*** like about the services that you received in the facility today?
9. Which services in this facility, would you want to be improved? Please tell me some more?

Appendix 9, consent form

CONFIDENTIAL

**Consent Form**

|  |  |
| --- | --- |

| **Study Title: Chronic respiratory symptoms in adults and children in Kenya: how do health systems respond and what are the opportunities for health system strengthening?** | |
| --- | --- |
| **Principal Investigator:** Stephen Kikwe Mulupi | **Study Site:** Kenya (Meru county) |

| **If you agree with each statement, please INITIAL the box provided** | |
| --- | --- |
| 1. I confirm I have read and understood the information sheet dated.................) for the above study. I have had the opportunity to consider the information, ask questions and have had these answered satisfactorily. |  |
| 1. I understand that participation in this study is voluntary and I am free to withdraw consent at any time, without giving a reason, without any penalties. |  |
| 1. I understand that data collected during the study may be looked at by individuals from or delegated by LSTM and from regulatory authorities. I give permission for these individuals to have access to my records. |  |
| 1. I hereby declare that I have not been subjected to any form of coercion in giving this consent. |  |
| 1. I agree to the data about me collected in this study being stored for further use in the future. |  |
| 1. I understand that my personal data will be handled in accordance with the applicable laws in Kenya, and the United Kingdom |  |
| 1. I understand that once my data has been fully anonymised, it cannot be deleted. |  |
| 1. I agree to take part in this study. |  |

Signing this declaration does not affect your right to decline to take part in any future study.

Name of participant Date Signature

Name of person taking Date Signature

Consent

When complete: 1 copy for participant; 1 copy (original) for research
