# Supplementary material for: “People here live in denial”: A qualitative study of the pervasive impact of stigma on asthma diagnosis and care in Kenya and Sudan
Source: PLOS Glob Public Health. 2025 Dec 16;5(12):e0003935. doi: 10.1371/journal.pgph.0003935 (PMC12707672; doi:10.1371/journal.pgph.0003935)
Supplement: Attachment — Submitted filename: Response to reviewers comments.docx [file pgph.0003935.s004.docx]

7^th^ May 2025

Re: response to reviewers’ comments

Dear Reviewers

Thank you for your useful comments. We believe we have addressed them all and that this has improved the paper. Please see the table below for a detailed response to each comment

Yours sincerely

Rachel Tolhurst, on behalf of all authors

| **Reviewer’s comment** | **Response** |
| --- | --- |
| **Abstract:** Should read ‘This contributes…’ | This has been amended |
| **Introduction** | |
| The introduction section is quite long for the reader, and it would benefit from a reduction in word count to provide a more succinct overview of the topic area. In particular, the definition/descriptions of stigma could be reduced, such as the Stangl framework, as this is discussed in the methods section. | The introduction has now been shortened, focusing on reduction of repetition of the Stangl framework |
| **Methods** | |
| It would be useful if the manuscript makes reference to the adherence to the COREQ reporting guideline [https://www.equator-network.org/reporting-guidelines/coreq/](https://url.uk.m.mimecastprotect.com/s/MS3uCPzkEi38mZ3c6t5cxbCIs?domain=equator-network.org) | This has been added |
| Final row Table 1 – Kenya, should read ‘convenience sampling’. | This has been amended |
| Line 189: the sentence needs wording to make clear that those dates were the pause dates. Perhaps ..COVID-19 restrictions (between 19/02/2020 and 15/07/2020), and in Sudan…’ | This change has been made |
| Are the topic guides available in the supplementary materials for focus group discussions? | These are now available in supplementary materials |
| Table 2 – IDI: acronym spell out first use | This change has been made |
| **Results** | |
| Figure 2 – Theme 3 – heading text ‘manifestations’ is across two lines | This has been amended |
| **Discussion** | |
| The discussion is also on the longer side and would benefit from a reduction in word count | The discussion has now been shortened |
| Lines 557-559 need a citation to back up this evidence. | A reference has been added |
| There are minor typos and spacing typos in the manuscript | These have been corrected |
